# Supplementary material for: Gender Differences in Protein Consumption and Body Composition: The Influence of Socioeconomic Status on Dietary Choices
Source: Foods. 2025 Mar 5;14(5):887. doi: 10.3390/foods14050887 (PMC11899518; doi:10.3390/foods14050887)
Supplement: Supplementary file 1 [file foods-14-00887-s001.zip › foods-3502919-supplementary.pdf]

Supplementary Material

Cereals and nuts consumption

The analysis of the participants' food preferences reveals some gender differences in the consumption of cereals and nuts (Figure S1). Women tend to state a greater preference for nuts than men, with a higher percentage of affirmative rather than reluctant or negative responses. In contrast, the distribution of preferences for cereals is more balanced between men and women. However, statistical tests did not confirm significant differences (p = 0.097 for cereals, p = 0.065 for nuts), suggesting that although there may be trends in preferences, these are not strongly driven by gender in this sample.

Figure S1. Gender Differences in Preferences for Cereals and Nuts

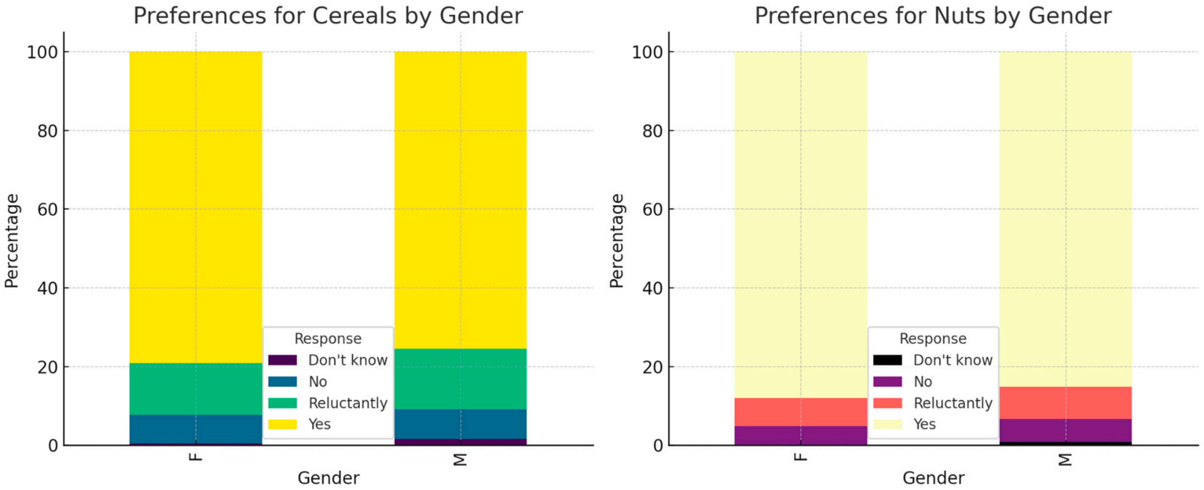

Figure S1. Preferences for cereals (left) and nuts (right) among men and women. Responses are expressed as a percentage and classified as Yes, Reluctant or No. The analysis reveals gender differences in food preferences, with women showing a greater preference for nuts, while cereals are more evenly distributed between genders. Chi-square tests indicate that these differences are not statistically significant (p = 0.097 for cereals, p = 0.065 for nuts).

–

Figure S2. Income-Based Differences in Preferences for Cereals and Nuts

The analysis of food preferences by income level suggests some trends in cereal and dried fruit consumption (Figure S2). Individuals in the lower income brackets tend to be slightly more likely to consume cereals and nuts, probably due to economic constraints and food availability. However, statistical tests did not confirm significant differences (p = 0.169 for cereals, p = 0.101 for nuts), indicating that income may not be a primary determinant of these preferences in this sample.

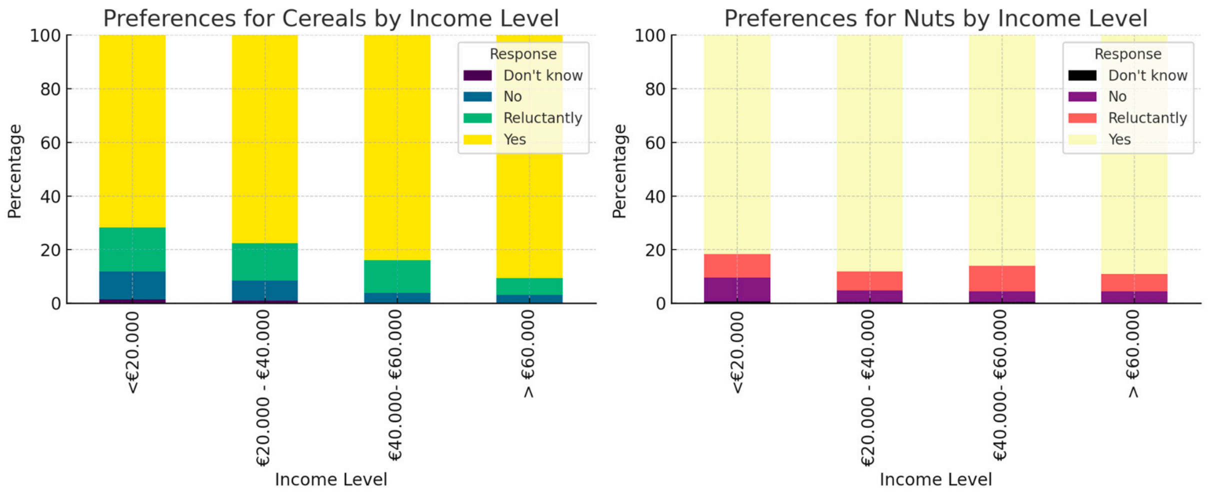

Figure S2.Preferences for cereals (left) and nuts (right) according to income level. Responses are classified as Yes, Reluctant or No and expressed as a percentage. The distribution suggests potential differences in food preferences according to income, with low-income individuals slightly more likely to consume cereals and nuts. However, Chi-square tests indicate that these differences are not statistically significant ($p = 0.169$ for cereals, $p = 0.101$ for nuts).

–

Study Limitations

This study has some limitations that need to be considered (Table S1).

Reliance on self-reported dietary data introduces potential recall and social desirability biases, which may have led to inaccuracies in food intake estimates. Variability in food composition, influenced by agricultural practices and processing methods, could also impact nutrient assessments, particularly for protein sources such as meat and dairy products. The cross-sectional design prevents causal inference, making it unclear whether dietary habits influence body composition or vice versa. Despite adjustments, the results may be influenced by unmeasured factors such as genetics, metabolic rate and physical activity. Finally, the generalisability of the results is limited by the study population, which may not fully reflect demographic and cultural variations in eating habits. Further research with longitudinal designs and more diverse samples is needed to strengthen these correlations and provide insights into gender-specific dietary patterns.

Despite the controls in place, the use of food diaries remains subject to potential recall and social desirability bias, especially with regard to certain food groups perceived as more or less healthy. Furthermore, the sample was recruited from a medical centre in Rome, which may limit the generalisability of the results to populations with different cultural backgrounds and food

availability. Although recruitment took place over almost two years, no specific data were collected on seasonal variations in diet, which could influence the frequency of consumption of certain foods.

A further limitation of the study concerns the collection of dietary data through 7-day food diaries, which are subject to recall and social desirability bias. Participants may have incorrectly reported the amount and type of food consumed, especially for those perceived to be more or less healthy. This phenomenon has been documented in previous studies, in which individuals tended to underestimate the intake of calorie-dense foods and overestimate the consumption of foods considered healthy [40].

To reduce these biases, participants received detailed instructions on accurate food recording, supported by visual material for standard portions. In addition, the diaries were reviewed by registered dietitians, who contacted the participants in case of inconsistencies to improve the accuracy of the data. However, other strategies, such as the use of food biomarkers or comparison with multiple food recall methods, could further improve the accuracy of dietary assessment in future studies [41].

One of the methodological limitations of this study concerns the use of bioimpedance analysis (BIA) for the assessment of body composition. Although BIA is widely used for its practicality, rapidity and low cost, it has certain limitations compared to gold standard methods such as dual-energy X-ray absorptiometry (DEXA). In particular, BIA is more sensitive to changes in hydration status, which can affect estimates of lean mass and fat mass [40]. In addition, BIA uses predictive equations based on specific populations, which could introduce biases into body composition estimates in individuals with particular physiological characteristics, such as athletes or people with severe obesity [41]. Comparative studies between BIA and DEXA have shown that although BIA offers good correlations with DEXA, it tends to underestimate body fat content in subjects with low levels of adiposity and overestimate it in subjects with high levels [42]. Therefore, the results of this study must be interpreted in light of these limitations. Future research could benefit from the combined use of more accurate methods, such as DEXA or air displacement plethysmography (BodPod), for a more precise assessment of body composition.

Another limitation concerns the composition of the sample, recruited in a medical centre in Rome, which could reduce the generalisability of the results to other populations with different dietary traditions. Finally, although recruitment took place over a period of almost two years, it was not possible to monitor in detail seasonal variations in diet, which may have influenced the frequency of consumption of certain protein sources.

Furthermore, some unmeasured confounding factors may have influenced the observed associations. Psychological factors such as stress, body image concerns and mental health conditions may have an impact on eating behaviour and physical activity levels, potentially altering gender-related protein intake patterns. For example, higher stress levels have been linked to higher consumption of ultra-processed high-energy foods, while body image concerns may lead to restrictive eating behaviour, especially among women. The lack of direct assessment of

these factors limits the ability to fully understand their role in shaping food choices. Future studies should incorporate validated psychological measures to better understand their influence on gender-specific eating behaviours.

Table S1. **Summary of Study Limitations**

Limitation	Description
**Self-reported dietary data**	Potential recall and social desirability biases may have led to inaccuracies in food intake estimates.
**Variability in food composition**	Differences in agricultural practices and food processing may impact nutrient assessments, particularly for protein sources like meat and dairy.
**Cross-sectional design**	Prevents causal inference, making it unclear whether dietary habits influence body composition or vice versa.
**Unmeasured factors**	Genetics, metabolic rate, and physical activity may have influenced results despite adjustments.
**Generalizability**	The study population may not fully reflect broader demographic and cultural variations in eating habits.
**Use of food diaries**	Recall and social desirability bias remain, especially for certain food groups perceived as more or less healthy.
**Recruitment site**	Participants were recruited from a medical center in Rome, which may limit the generalizability of findings to different cultural and dietary contexts.

**Seasonal dietary variation**	No data were collected on seasonal dietary changes, which may have influenced food intake patterns.
**Body composition assessment**	BIA, while practical and widely used, has limitations compared to gold-standard methods like DEXA and is influenced by hydration status.
**Psychological factors**	Stress, body image concerns, and mental health conditions, which may affect eating behaviors, were not directly assessed.
